# Upregulation of cell surface GD3 ganglioside phenotype is associated with human melanoma brain metastasis

**DOI:** 10.1002/1878-0261.12702

**Published:** 2020-06-05

**Authors:** Romela Irene Ramos, Matias A. Bustos, Jinfeng Wu, Peter Jones, Shu Ching Chang, Eiji Kiyohara, Kevin Tran, Xiaoqing Zhang, Stacey L. Stern, Sivan Izraely, Orit Sagi‐Assif, Isaac P. Witz, Michael A. Davies, Gordon B. Mills, Daniel F. Kelly, Reiko F. Irie, Dave S. B. Hoon

**Affiliations:** ^1^ Department of Translational Molecular Medicine John Wayne Cancer Institute (JWCI) Santa Monica CA USA; ^2^ Department of Dermatology Huashan Hospital Fudan University Shanghai China; ^3^ Medical Data Research Center Providence St. Joseph Health Center Portland OR USA; ^4^ Department of Biostatistics JWCI Santa Monica CA USA; ^5^ Department of Cell Research and Immunology George S. Wise Faculty of Life Sciences Tel‐Aviv University Tel Aviv Israel; ^6^ Department of Melanoma Medical Oncology Systems Biology and Translational Molecular Pathology The University of Texas MD Anderson Cancer Center Houston TX USA; ^7^ Department of Cell Development and Cancer Biology Oregon Health and Science University (OHSU) Knight Cancer Institute Portland OR USA; ^8^ Pacific Neuroscience Institute JWCI Santa Monica CA USA

**Keywords:** gangliosides, GD3, lymph node, melanoma brain metastasis, NF‐κB, ST8SIA1

## Abstract

Melanoma metastasis to the brain is one of the most frequent extracranial brain tumors. Cell surface gangliosides are elevated in melanoma metastasis; however, the metabolic regulatory mechanisms that govern these specific changes are poorly understood in melanoma particularly brain metastases (MBM) development. We found ganglioside GD3 levels significantly upregulated in MBM compared to lymph node metastasis (LNM) but not for other melanoma gangliosides. Moreover, we demonstrated an upregulation of ST8SIA1 (GD3 synthase) as melanoma progresses from melanocytes to MBM cells. Using RNA‐ISH on FFPE specimens, we evaluated ST8SIA1 expression in primary melanomas (PRM) (*n* = 23), LNM and visceral metastasis (*n* = 45), and MBM (*n* = 39). ST8SIA1 was significantly enhanced in MBM compared to all other specimens. ST8SIA1 expression was assessed in clinically well‐annotated melanoma patients from multicenters with AJCC stage III B‐D LNM (*n* = 58) with 14‐year follow‐up. High ST8SIA1 expression was significantly associated with poor overall survival (HR = 3.24; 95% CI, 1.19–8.86, *P* = 0.02). In a nude mouse human xenograft melanoma brain metastasis model, MBM variants had higher ST8SIA1 expression than their respective cutaneous melanoma variants. Elevated ST8SIA1 expression enhances levels of cell surface GD3, a phenotype that favors MBM development, hence associated with very poor prognosis. Functional assays demonstrated that ST8SIA1 overexpression enhanced cell proliferation and colony formation, whereby ST8SIA1 knockdown had opposite effects. Icaritin a plant‐derived phytoestrogen treatment significantly inhibited cell growth in high GD3‐positive MBM cells through targeting the canonical NFκB pathway. The study demonstrates GD3 phenotype associates with melanoma progression and poor outcome.

## Graphical abstract

We demonstrated the upregulation of a ganglioside synthase, ST8SIA1 and GD3 expression in MBM, which can be suppressed by icaritin treatment. Icaritin inhibits NFκB‐mediated activation of ST8SIA1 by downregulating p50. These findings suggest that ST8SIA1 is a significant factor and potential target to MBM.

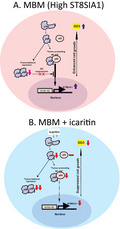

**Abbreviations**
Ab: antibodies
DOM: distant organ metastasis
FACS: fluorescence‐activated cell sorting
FFPE: formalin‐fixed paraffin‐embedded
JWCI: John Wayne Cancer Institute
KD: knockdown
LNM: lymph node metastasis
mAb: monoclonal antibodies
MBM: melanoma brain metastasis
MNCs: melanocytes
NS: not significant
OS: overall survival
PRM: primary melanomas
RNA‐ISH: RNA *in situ* hybridization
ST8SIA1‐OV: ST8SIA1 overexpression
WIRB: Western Institutional Review Board

## 1 Introduction

Melanoma is an aggressive cancer with increasing incidence worldwide (George *et al*., 2016), and the propensity to metastasize frequently to multiple organ sites contributes to its poor prognosis. Cutaneous melanoma has the highest frequency of brain metastasis among all extracranial primary origin tumors (Izraely *et al*., 2019). While treatments for early‐stage lymph node and organ metastasis have improved, the mortality and morbidity related to melanoma brain metastasis (MBM) still remain a major clinical problem, and survival two years postdiagnosis is dismal (Choong *et al*., 2017; Davies *et al*., 2017; Eichler *et al*., 2011; Sloot *et al*., 2018). Beyond early‐stage elective surgery, effective treatments for MBM with immune checkpoint inhibitors and targeted therapy have shown better durable responses (Tawbi *et al*., 2018a). Recently, we have identified several factors facilitating MBM progression (Bustos *et al*., 2017; Iida *et al*., 2017; Izraely *et al*., 2017, 2019; Klein *et al*., 2017; Marzese *et al*., 2015; Orozco *et al*., 2018; Prakash *et al*., 2019; Sloot *et al*., 2018; Tawbi *et al*., 2018b); however, no single genomic mutation or epigenomic aberration has been found to be significantly associated with MBM development (Orozco *et al*., 2018), suggesting the involvement of a more complex mechanisms other than genomic aberrations.

The predominant cell surface gangliosides in human cutaneous melanomas are GM3 and GD3 (Tsuchida *et al*., 1987b). The precursor ganglioside GM3 is the major cell surface ganglioside present on melanocytes, nevi, and primary melanomas (PRM) and is converted to GD3 during progression (Carubia *et al*., 1984; Ravindranath *et al*., 1991). The metabolic ganglioside pathways leading to the biosynthesis of the three major cell surface gangliosides GM2, GD3, and GD2 in cutaneous melanomas are significantly altered during melanoma development and progression (Ravindranath *et al*., 1991; Tsuchida *et al*., 1989). ST8SIA1 (also referred to as GD3 synthase) is the rate‐limiting enzyme for GD3 synthesis from the precursor ganglioside GM3, while β4GALNT1 is the key enzyme responsible for GM2 and GD2 synthesis from precursor gangliosides GM3 and GD3, respectively (Groux‐Degroote *et al*., 2017). Specific sialyltransferases, which add sialic acid to the individual ganglioside sugars to develop specific disialic gangliosides such as GD3 and GD2, have been described previously (Tsuchida *et al*., 1989). In this study, our observation of significant changes in ganglioside metabolism during melanoma progression to MBM implies that activation of specific ganglioside pathways is a major factor promoting this metastasis progression. However, little is known on the role of ST8SIA1, the key enzyme for GD3 expression leading to MBM development.

We *hypothesized* that ST8SIA1 plays a role in MBM development by promoting GD3 phenotypic expression though the activation of the canonical NF‐κB pathway. Our results demonstrated that ST8SIA1 upregulation favors metastatic melanoma progression in lymph node metastasis (LNM) and MBM development; moreover, we demonstrated that high ST8SIA1 expression is a significant independent prognostic factor for LNM patients in multivariable analysis.

We identified a potential inhibitor drug of ST8SIA1 expression, which is icaritin. This drug is a herbal medicine extract derived from the plant *Herba epimedeii* with antimelanoma cell inhibitory activity as we previously shown (Wu *et al*., 2016). Icaritin is a phytoestrogen that has structural similarity to estradiol. Icaritin suppresses melanoma growth and modulates fatty acids of cell membranes as we previously described (Wu *et al*., 2016). Therefore, we have investigated the effect of icaritin on ganglioside synthesis regulation in melanoma cells as cell surface‐specific membrane gangliosides are significantly elevated in melanoma progression. Since icaritin is currently in clinical trials for metastatic solid tumors (clinicaltrials.gov), we further pursued its effect as a preclinical *in vitro* study in metastatic cutaneous melanoma cells.

In this study, we discovered that a plant derivative natural compound icaritin targets particularly high GD3 expressing MBM cells and downregulates ST8SIA1, by decreasing the canonical NF‐κB pathway activation, resulting in reduced cell growth and colony cell formation.

## 2 Materials and methods

### 2.1 Antibodies and reagents

Purified icaritin (> 99% pure) was assessed (Shanghai Winherb Pharmaceutical Co., Ltd. Shanghai, China). For treatment of cells, the dosage was determined from previous studies with icaritin (Wu *et al*., 2016). Antibodies (Ab) used in fluorescence‐activated cell sorting (FACS) staining were as follows: FITC‐conjugated mouse monoclonal Ab (mAb) anti‐GD2 (Clone 14.G2a, BD Biosciences, San Jose, CA, USA, Cat# 563439); unconjugated mouse mAb anti‐GD3 (Clone R24, Abcam, Cambridge, MA, USA, Cat# ab11779) with secondary polyclonal Ab goat anti‐mouse IgG3‐APC‐Cy7 (Abcam Cat# ab47000); and purified human monoclonal mAbs IgM anti‐GM2 (L55) and IgM anti‐GM3 (L612) were produced and purified by R. Irie (Hoon *et al*., 1992; Irie *et al*., 2004). The secondary Ab used was mouse mAb anti‐human IgM‐FITC (Clone G20‐127, BD Biosciences Cat# 562029). 7‐Amino‐actinomycin D (7‐AAD) Ab type (BD Biosciences, Cat# 559925) was used for dead cell discrimination. For the GM2 and GM3 IgM mAb, 20 and 30 µg·mL^−1^ were optimized, respectively, for FACS. Abs used in western blotting were as follows: mouse mAb anti‐p50 (Santa Cruz Biotechnology, Dallas, TX, USA, Cat# sc‐8414), mouse mAb anti‐p65 (Santa Cruz Biotechnology Cat# sc‐8008), rabbit polyclonal IgG purified anti‐ST8SIA1 (Proteintech, Rosemont, IL, USA, Cat# 24918‐1‐AP), mouse mAb anti‐β‐actin (Clone AC‐15, Sigma‐Aldrich, St. Louis, MO, USA, Cat# A5441), horseradish peroxidase‐conjugated Abs: sheep polyclonal purified IgG anti‐mouse Ab (GE Healthcare, Pittsburgh, PA, USA, Cat# NA931) or donkey polyclonal purified IgG anti‐rabbit Ab (GE Healthcare, Cat# NA934). Abs used in immunofluorescence staining of cell lines are mouse mAb anti‐GD3 (Clone R24, Abcam, Cat# ab11779) and goat polyclonal IgG anti‐mouse Ab (Alexa Fluor^®^ 594) (Jackson ImmunoResearch, Westgrove, PA, USA, Cat# 115‐585‐003). Abs concentrations were based on optimization and/or manufacturer's recommendations.

### 2.2 Cells and tissues

In genomewide gene expression profiling in metastatic melanoma cell lines using Affymetrix HuEx 1.0 ST array (GSE44662) (*n* = 50), we used melanocytes (MNCs) (gift from L. Garraway, Broad Institute, Cambridge, MA, USA) as previously described (Lessard *et al*., 2015). MNCs were used as a negative control for determining the ST8SIA1 expression in LNM and MBM cells. All metastatic melanoma cell lines were cultured in RPMI‐1640 (Corning, Manassas, VA, USA) with 10% heat‐inactivated fetal bovine serum and antibiotic and then mRNA extracted as previously described (Bustos *et al*., 2017). We assessed 107 formalin‐fixed paraffin‐embedded (FFPE) melanoma tumors (PRM, LNM, MBM, and distant metastasis). FFPE tissues were obtained from the Division of Surgical Pathology, SJHC. In addition, we assessed tissues procured from Division of Surgical Pathology and stored in the JWCI liquid nitrogen cryobank: 10 MBMs, 5 meningiomas, and 5 benign pituitary adenomas. Meningiomas and pituitary adenomas frozen tissues were used as a control for MBM frozen tissue analysis of ST8SIA1 mRNA expression. This study followed the principles in the Declaration of Helsinki. Experiments were undertaken with the understanding and written consent of each subject. All human samples and clinical information for this study were obtained according to the protocol guidelines (protocol numbers: JWCI‐18‐0401 and MORD‐RTPCR‐0995) approved by the Providence Health and Services IRB and Western Institutional Review Board (WIRB).

### 2.3 Overexpression and knockdown of ST8SIA1 gene

M16, ML‐0817, and M‐204 cells (0.3 × 10^6^ cells/well) were seeded in 6‐well culture plates (Corning, NY, USA) and transfected with T7‐tagged pReceiver‐M98 vector encoding ST8SIA1 (GeneCopoeia, Rockville, MD, USA) using jetPRIME (Polyplus Transfection, New York, NY, USA). ST8SIA1 overexpression was evaluated using quantitative reverse transcriptase/polymerase chain reaction (qRT‐PCR). DP‐0574 and M‐204 cells (0.3 × 10^6^/well in 6‐well culture plates) were transfected with 10 nm ON‐TARGETplus Human ST8SIA1 siRNA SMARTpool (#L‐011775‐01‐0005; Dharmacon, Thermo Fisher Scientific, Carlsbad, CA, USA) and ON‐TARGETplus nontargeting pool (#D‐001810‐10‐05; Dharmacon) using jetPRIME (Polyplus Transfection). ST8SIA1 expression was evaluated 48 h after transfection by qRT‐PCR. For colony formation assay, transfected cells were seeded (2000 cells/well in a 6‐well culture plate) and incubated for 12 days. Colonies were stained with crystal violet and quantified as previously described (Bustos *et al*., 2017).

### 2.4 3D culture

Vitrogel 3D culture (Well Bioscience Inc., Newark, NJ, USA) was utilized to mimic the tumor environment. Hydrogel was diluted with distilled water in a 1 : 3 ratio and mixed 1 : 1 with a suspension of cells in RPMI treated or untreated with icaritin (40 µm); then, 0.1 × 10^6^ cells per 250 µL were seeded per well in a 24‐well culture plate (Corning Inc., Corning, NY, USA). Cells in the hydrogel solution were incubated for 15 min at RT, and RPMI medium was added on top of the gel before incubation at 37 °C. After 72 h, cells were harvested and were used for downstream assays (FACS, qRT‐PCR) to compare 2D with 3D cultures.

Cells were grown in 96‐well flat‐bottom culture plates (Thomas Scientific, Swedesboro, NJ, USA) and 96‐well spheroid culture plates (Corning, Inc.) treated with or without icaritin (40 µm) to measure cell proliferation. Light microscopy images of spheroid cultures were taken at days 1 and 3 at 4× magnification. After 72 h of culture, the number of viable cells was determined using the CellTiter‐Glo Luminescent Cell Viability Assay (Promega, Madison, WI, USA) as previously described (Bustos *et al*., 2017).

### 2.5 FACS analysis of cell surface ganglioside expression

MBM and LNM cell lines at 70% confluence were treated for 72 h with icaritin or left untreated. After 72 h, the cells were washed with cold PBS (pH 7.4) and detached using a nonenzymatic cell stripper (Corning, Cat# 25‐056‐CI). The single‐cell suspension was centrifuged (300 ***g***), washed with PBS, stained with primary mAbs (GM2, GM3, GD2, and GD3), and incubated for 20 min RT in the dark, washed with FACS buffer (Thermo Fisher Scientific, Cat# 00‐4222‐57), and centrifuged (300 ***g***). Cells were stained with secondary Ab and incubated for 20 min at RT in the dark. After washing with staining buffer, cells were resuspended in 200 µL of staining buffer and 10 µL of 7‐AAD Ab (viability analysis) (BD Bioscience, Cat# 559925) was added to differentiate live from dead cells. Data of 10 000 events were acquired on a FACSVerse (BD Bioscience). Data analysis was performed using flowjo software V10 (Tree Star, Ashland, OR, USA).

### 2.6 Real‐time PCR assay

Total cellular RNA was extracted using ZR‐Duet DNA/RNA MiniPrep kit (Zymo Research, Irvine CA, USA), and quantified and assessed for purity as previously described (Iida *et al*., 2017). Reverse transcription was performed to obtain cDNA using M‐MLV reverse transcriptase (PAM1705; VWR, Radnor, PA, USA), according to the manufacturer's protocol. The primers were synthesized by Integrated DNA Technologies (San Diego, CA, USA). The ST8SIA1 primer sequences were as follows: 5′‐ATTGAAGAAATGCGCGGTGGT‐3′ (forward); 5′‐GAGGGAGATTGCATCGCATGA‐3′ (reverse). The β4GALNT1 primers were as follows: 5′‐GATGTTGTACTGGGCTCCCT‐3′ (forward); 5′‐CCAACACAGCAGACACAGTC‐3′ (reverse). The SDHA (succinate dehydrogenase complex, subunit A) primer sequences were as follows: 5′‐TCAGCATGCAGAAGTCAAT‐3′ (forward); 5′‐GAACGTCTTCAGGTGCTTT‐3′ (reverse). Expression of the housekeeping gene, SDHA, served as an internal reference for mRNA integrity (Iida *et al*., 2017). qRT‐PCR was performed using SYBR Green reaction mixture (Quanta Bio, Beverly, MA, USA).

### 2.7 RNA *in situ* hybridization

Cells (0.2 × 10^5^) were seeded into 8‐well culture chamber slides and incubated for 24 h. Cells were fixed with neutral and buffered formalin (1 : 10) (#575A‐1gl; Medical Chemical Corporation, Torrance, CA, USA), dehydrated, and pretreated according to the manufacturer's instructions. Cells were then stained with the Hs‐ST8SIA1 probe (#498361) using the RNAscope Multiplex Fluorescent Kit V2 (ACD, Newark, CA, USA). Immunofluorescence staining was then observed under a fluorescence microscope, and representative images were taken for each condition. Images were analyzed based on ACD guidelines for ISH scoring. Assays for FFPE tissues were developed and optimized as previously described (Hooda *et al*., 2018).

### 2.8 Western blot analysis

For total protein extractions, cells subjected to icaritin treatment for 72 h or control conditions were harvested and resuspended in cell extract buffer with protease inhibitor (Thermo Fisher Scientific); whole‐cell extracts were incubated and centrifuged, and lysates were frozen at −30 °C. The protein concentration was determined using the bicinchoninic acid (BCA) protein assay kit (Thermo Fisher Scientific). Twenty micrograms of cellular protein was separated on a 10% SDS/PAGE and electroblotted onto a PVDF membrane (GE Healthcare). The membrane was blocked, incubated with primary and secondary Abs, and developed as previously described (Iida *et al*., 2017).

### 2.9 Stable clones overexpressing ST8SIA1

To establish stable clones with ST8SIA1‐OV, DP‐0574 and M‐204 cells (5 × 10^3^ in 24‐well plates) were transduced with ST8SIA1 vector using lentivirus particles (Cat#:LPP‐C0670‐Lv205‐050; GeneCopoeia, Rockville, MD, USA) and control particles coding for the empty vector (Cat#: LPP‐NEG‐Lv205‐050; GeneCopoeia). Positive clones were selected using Puromycin (0.4 µg·mL^−1^, Cat: A1113803, Life Technologies, Carlsbad, CA, USA) and ST8SIA1‐OV was confirmed by western blot. All experiments that involved clones with ST8SIA1‐OV were performed within ten passages after their establishment.

### 2.10 Immunofluorescence

Cells (1–1.5 × 10^4^) were seeded in 8‐well culture slides (Falcon™ Chambered Cell Culture Slides, 08‐774‐26, Fisher Scientific, Hampton, NH, USA). The cells were incubated for 48 h. After that, cells were fixed and the protocol was followed as previously described (Bustos *et al*., 2017). The photographs were obtained using a Leica SP8 Confocal microscope (Leica, Wetzlar, Germany).

### 2.11 Cell transactivation assay

Nuclear extracts from cell lines with or without icaritin (40 µm) treatment were isolated using Nuclear Extract Kit according to the manufacturer's instructions (#40010; Active Motif, Carlsbad, CA, USA). Isolated nuclear extracts were stored at −30 °C until used. Five micrograms of nuclear extract was used to detect and quantify p50 activation using a transactivation assay (TransAM NF‐κB p50 kit; #41096; Active Motif).

### 2.12 Cutaneous and MBM variants established in a mouse xenograft model

We developed tumors from YDFR human melanoma cell line using male athymic nude‐BALB/c. From these tumors, we established cutaneous variants (C) and MBM variants (CB3) that went to these specific organ sites when injected to the mice as previously described (Izraely *et al*., 2017, 2012). RNA was isolated from FFPE tumor blocks or resected tumors obtained from two pairs of cutaneous variants and MBM variants from the human parental YDFR cell line (Izraely *et al*., 2012), and ST8SIA1 mRNA expression was evaluated by qRT‐PCR.

### 2.13 Biostatistical analyses

Continuous variables were assessed using Wilcoxon rank‐sum tests or Student's *t*‐test (2‐tailed) or Welch's unequal variances *t*‐test. All data are expressed as means ± SD (standard deviation) or SEM (standard error of the mean) where indicated. The correlation of ST8SIA1 expression to the cell surface GM3 and GD3 expression was plotted using r package ‘corrplot’. Overall survival (OS), adjusted for age, gender, and AJCC 8 stage III B‐D categories, was analyzed using Kaplan–Meier analysis and log‐rank test, and multivariable survival analysis was performed by Cox proportional regression model. ST8SIA1 expression was divided into low and high groups based on the optimal cutoff values determined by the maximally selected rank statistics using ‘surv_cutpoint’ function of statistical r package ‘survminer’ (Lesueur *et al*., 2018; Zeng *et al*., 2018). All statistical analyses were performed using r version 3.5.0 (R Core Team, 2018). A *P*‐value < 0.05 was regarded as statistically significant.

## 3 Results

### 3.1 ST8SIA1 expression is enhanced in MBM cell lines

**Fig. 1:**
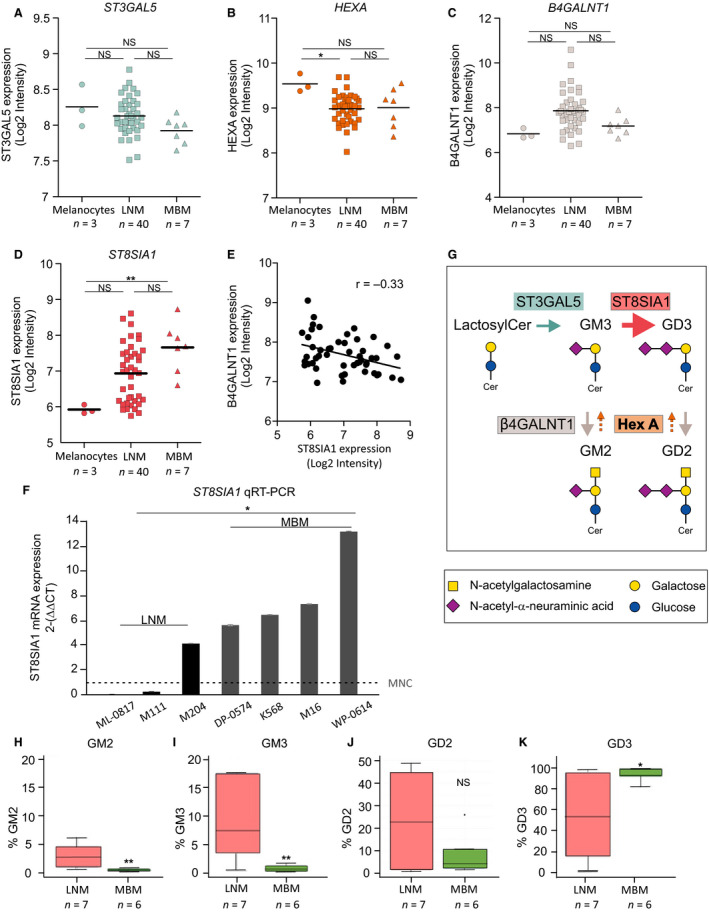
Enzymes involved in the synthesis of specific gangliosides in MBM. (A–D). mRNA expression of ST3GAL5 (A), HEXA (B), B4GALNT1 (C), and ST8SIA1 (D) assessed by genomewide exon array in cell lines [melanocytes (*n* = 3), LNM (*n* = 40), and MBM (*n* = 7)]. (E). Negative correlation between ST8SIA1 and B4GALNT1 in cell lines (*n* = 50). (F). ST8SIA1 mRNA expression ( 2 ${}^{-(\Delta \Delta C_{T})}$ ${}^{-(\Delta \Delta C_{\text{T}})}$ values) was assessed in cell lines [melanocytes (*n* = 1), LNM (*n* = 3), and MBM (*n* = 4)] by qRT‐PCR. (G). Biosynthesis pathway of the gangliosides (GM3, GM2, GD3, and GD2) and the enzymes (ST3GAL5, ST8SIA1, β4GALNT1, and HEXA) involved are shown. (H–K). Analysis of the phenotypic cell surface expression of GM2 (H), GM3 (I), GD2 (J), and GD3 (K) in LNM (*n* = 7) and MBM (*n* = 6) assessed by FACS. Cells were gated for live cell population using 7‐AAD dye and evaluated for GM2‐, GM3‐, GD2‐ and GD3‐positive expression. Box plots represent the distribution of the percentage of live cells expressing GM2, GM3, GD2, and GD3, respectively. (*t*‐test, *P < 0.05, **P < 0.01).

Specific cell surface gangliosides are overexpressed during transformation from melanocytes to advance stages of metastasis (Tsuchida *et al*., 1987b); however, the expression of specific enzymes part of the pathways' biosynthesis in MBM is unknown. Therefore, we analyzed the expression of different genes for key ganglioside enzymes involved in melanoma ganglioside metabolism, ST3GAL5 (CMP‐Neu5Ac:lactosylceramide α2,3‐sialyltransferase; GM3 synthase), which catalyzes the formation of GM3 using lactosylceramide as the substrate), HEXA (hexosaminidase α subunit, which converts GM2 and GD2 into GM3 and GD3, respectively), B4GALNT1 (β1,4‐*N*‐acetylgalactosaminyltransferase 1; GM2/GD2 synthase, which converts GM3 and GD3 into GM2 and GD2, respectively), and ST8SIA1 (CMP‐Neu5Ac:GM3 α2,8‐sialyltransferase; GD3 synthase, which converts the precursor ganglioside GM3 to GD3). Using genomewide gene expression (Affymetrix HuEx 1.0 ST array) profiling (GSE44662) (*n* = 50), ST3GAL5, HEXA, and B4GALNT1 did not show significant differences in MNC, LNM, and MBM (Fig. 1A–C); however, ST8SIA1 was significantly increased in MBM compared to MNC (Fig. 1D). Of note, we observed LNM cells varied from high to low ST8SIA1 gene expression. Also, we observed significant negative correlation between ST8SIA1 and B4GALNT1 expression in different sites: melanocytes, LNM, and MBM (Fig. 1E). Furthermore, ST8SIA1 mRNA expression was validated in LNM and MBM cell lines by qRT‐PCR analysis using MNC as a reference control. MBM but not LNM cells showed significant increase of ST8SIA1 expression compared to MNC cells. Consistently, MBM cells had significantly higher expression of ST8SIA1 than LNM cells (Fig. 1F). RNA in situ hybridization (RNA‐ISH) assays confirmed higher ST8SIA1 mRNA expression in MBM compared to LNM cell lines (Fig. S1A). As summarized in Fig. 1G (four main melanoma ganglioside biosynthesis following the official recent nomenclature) (Neelamegham *et al*., 2019; Varki *et al*., 2015), we observed that all four enzymes (described above) involved in ganglioside synthesis were expressed variably in the metastatic melanoma cell lines, but only ST8SIA1 expression was elevated in MBM cells, suggesting the mechanistic role in increasing GD3 expression levels.

### 3.2 GD3 is the predominant cell surface ganglioside expressed in MBM

To confirm our ganglioside expression findings above, we performed FACS analysis using specific antiganglioside mAbs to compare individual ganglioside expression in regional primary tumor‐draining LNM and MBM cell lines. GM3 was absent or was limited in MBM cells, whereas GM2 was undetectable in MBM cells. GM3 and GM2 showed significantly lower expression in MBM compared to LNM cell lines (Fig. 1H,I). GD2, a disialic acid ganglioside, was minimally expressed and showed no differences between MBM and LNM cells (Fig. 1J). GD3, also a disialic acid ganglioside, was significantly higher overall on MBM cells compared to LNM cells (Fig. 1K). We categorized GD3 levels on melanoma cell lines as high to moderate (50% to > 90% positive) and low (< 50% positive) as a relative level of expression by FACS in all further experiments as a reference of the cell lines used. Overall, we observed an elevated GD3 expression on the cell surface of MBM cell lines, whereas the expression of GM3, GM2, and GD2 was significantly lower or absent.

We assessed correlation between ST8SIA1 expression and GD3 or GM3 levels in all the melanoma cell lines profiled. There was a positive correlation between ST8SIA1 and GD3 expression (*r* = 0.90), but a negative correlation with GM3 (GD3 precursor) expression (Fig. S1B). These results suggested that ST8SIA1 plays a significant role in enhancing the levels of cell surface GD3 in MBM cells.

### 3.3 ST8SIA1 is highly expressed in clinical MBM tumor samples

**Fig. 2:**
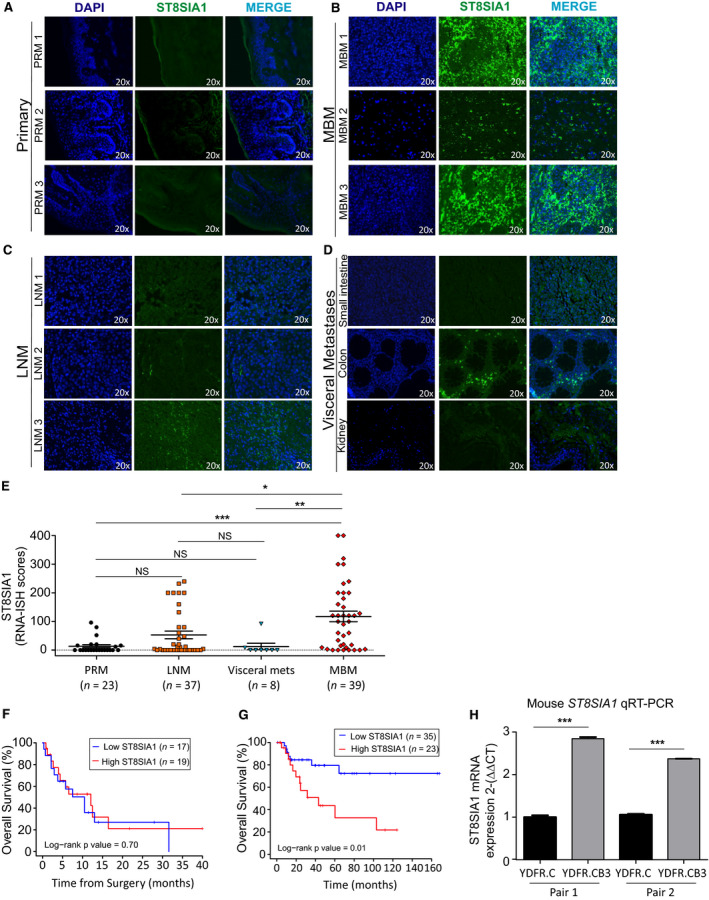
Profile and prognostic values of ST8SIA1 expression in LNM and MBM. (A–E). FFPE tissue sections from melanoma patients were stained with ST8SIA1 probe for RNA‐ISH and assessed by immunofluorescence. Representative images are shown for ST8SIA1 expression; DAPI (blue), ST8SIA1 (green), and Merge in PRM (A), MBM (B), LNM (C), and various visceral metastases (small intestine, colon, and kidney) (D). (E). ST8SIA1 RNA‐ISH scores (0–400) in PRM (*n* = 23), LNM (*n* = 37), visceral metastasis (*n* = 8), and MBM (*n* = 39). (F). Kaplan–Meier curve comparing OS for MBM patients with high and low ST8SIA1 expression based on median cutoff of RNA‐ISH scores. (G). Kaplan–Meier curve comparing OS for patients with high and low ST8SIA1 expression (*z*‐score cutoff = 0.31, *n* = 58). (H). qRT‐PCR analysis of ST8SIA1 expression in paired tissue from PRM tumors (YDFR.C) and MBM (YDFR.CB3) derived from xenograft mouse model. Error bars represent means ± SD of three replicates (*t*‐test; NS: not significant, *P < 0.05, **P < 0.01, ***P < 0.001).

Unfortunately, pathology tissue processing with formalin fixation destroys gangliosides on the cell surface membrane; therefore, IHC staining of FFPE tumor sections is not useful. To determine the translational relevance of our findings, RNA‐ISH was performed in FFPE melanoma tissue specimens using our specifically designed ST8SIA1 mRNA detection probe. All the tissues were quantified for staining to calculate the RNA‐ISH score. Respective positive and negative control probes were performed on respective section used. MBMs (*n* = 39) showed significant higher expression of ST8SIA1, compared to PRM (*n* = 23), LNM (*n* = 37), and visceral metastases (*n* = 8) (Fig. 2A–E; *n* = 107). Moreover, we verified the FFPE MBM findings by assessing ST8SIA1 expression by qRT‐PCR in clinicopathological annotated frozen tissues: MBM, *n* = 10; and benign tissues such as pituitary adenomas; *n* = 5, and meningioma; *n* = 5. Concordantly, MBM frozen samples had significantly higher ST8SIA1 expression compared to the benign frozen tissues assessed (Fig. S1C). Furthermore, we evaluated the association between ST8SIA1 expression and clinical/pathology demographics using the melanoma TCGA database; however, it is important to mention that we found no clinically annotated MBM in TCGA melanoma database (version 2019). The majority of the TCGA melanoma specimens are LNM and then followed by PRM and some distant organ site metastasis (DOM). Using the TCGA melanoma dataset, we observed no differences in ST8SIA1 expression when comparing PRM, LNM, and DOM as it was low and variable in general (Fig. S2A). However, we compared PRM melanoma with high or low ST8SIA1 expression to determine associations with standard prognostic clinical and pathology factors. We did not observe association between AJCC Stage (8th ed) (Fig. S2B), primary melanoma prognostic factors such as ulceration (Fig. S2C), Breslow depth (Fig. S2D), or age at diagnosis (Fig. S2E), and ST8SIA1 expression. However, we observed a significant higher ST8SIA1 expression in melanoma patients with BRAF V600e mutation compared to those that were triple WT (wild type of BRAF, NRAS, and NF1 mutations) or NRAS mutation(s) alone (Fig. S2F).

Analyzing clinical outcomes in MBM patients is not very feasible because in general they have a dismal prognosis with a short‐term survival once diagnosed, variable stage at time of diagnosis, and receive multiple modality treatment regimens that fail. We utilized the RNA‐ISH scores of MBM patients from JWCI with available clinical outcomes (*n* = 36) to split patients into high and low ST8SIA1 expression and assessed OS. As expected, there was no obvious ST8SIA1 relation to OS in MBM patients (Fig. 2F) due to multiple clinical variables such as the number of metastasis, sites, and location, which are significant factors in OS.

We then performed analysis on patients with LNM because of the differential expression of ST8SIA1 and the availability of long‐term clinical follow‐up available in the TCGA melanoma dataset in a multicenter setting. The identification of high‐risk factors in LNM predicting recurrence after regional surgical LN resection to render disease‐free status remains an important clinical management issue as we and others have shown (Ecker *et al*., 2018; Ekmekcioglu *et al*., 2016; Faries *et al*., 2017; Han *et al*., 2013). Thus, we assessed the association between ST8SIA1 expression in LNM and OS by using the TCGA cohorts of stage III B‐D melanoma patients (corrected AJCC 8th ed) with clinically annotated 14‐year follow‐up data and annotated clinicopathologically (*n* = 58, Table 1). Kaplan–Meier curves demonstrated that patients with LNM and high ST8SIA1 expression were associated with significantly shorter OS than those with low ST8SIA1 expression (Fig. 2G). Importantly, multivariable analysis of known prognostic factors including age, gender, and AJCC stage III B‐D status revealed that high ST8SIA1 expression was associated with poor OS (HR 3.24; 95% CI, 1.19–8.86, *P* = 0.02; Table 2). Also, this finding suggested that high ST8SIA1 expression in patients with stage III B‐D LNM is an independent prognostic factor for poor outcome; moreover, those patients have a high risk to distant organ metastasis including the brain.

**Table 1:** Patient characteristics.

Age, mean (SD)	54.95 (14.78)
Gender (%)	
Female	25 (43.1)
Male	33 (56.9)
AJCC stage (8th ed)	
IIIB	20 (34.5)
IIIC or D	38 (65.5)

**Table 2:** Univariable and multivariable cox proportional regression analysis for the association of ST8SIA1 expression with OS.

	Univariable analysis				Multivariable analysis			
	HR	Lower	Upper	*P* value	HR	Lower	Upper	*P* value
		95% CI	95% CI			95% CI	95% CI	
ST8SIA1^a^								
Low	1				1			
High	3.20	1.25	8.18	**0.02**	3.24	1.19	8.86	**0.02**
Age at diagnosis	1.02	0.99	1.06	0.16	1	0.97	1.04	0.90
Gender								
Female	1				1			
Male	0.56	0.23	1.38	0.21	0.42	0.15	1.19	0.10
AJCC stage (8th ed)								
IIIB	1				1			
IIIC/D	1.85	0.67	5.15	0.24	2.68	0.85	8.41	0.09

^a^ ST8SIA1 low and high groups were based on optimal cutoff determined by the maximally selected rank statistics.

Bold‐faced values indicate significant *P* values.

### 3.4 ST8SIA1 expression of human MBMs in mouse xenografts

We have shown that certain human melanoma tumor xenografts have better brain metastasis growth success in nude mice (Tsuchida *et al*., 1987a). We assessed our well‐established nude mouse human MBM variant xenograft models for ST8SIA1 expression (Izraely *et al*., 2017; Klein *et al*., 2017). Briefly, in the xenograft nude mice model, the cutaneous melanoma variants were intracardially injected until they spontaneously developed MBM (Izraely *et al*., 2017; Klein *et al*., 2017). To determine whether ST8SIA1 was differentially expressed in brain‐metastasizing melanoma cell variant (YDFR.CB3) compared to the respective cutaneous variant (YDFR.C). We performed qRT‐PCR on the tumors, resected at the respective organ site, developed after each variant type was injected into the mouse. The brain‐metastasizing melanoma cell variant (YDFR.CB3) expressed higher mRNA levels of ST8SIA1 than cells from the corresponding parental cutaneous variant (YDFR.C) (Fig. 2H). Thus, this model further supported that ST8SIA1 expression is enhanced in MBM, suggesting a role for ST8SIA1 in MBM establishment and progression.

### 3.5 ST8SIA1 overexpression enhances cell proliferation and colony formation

**Fig. 3:**
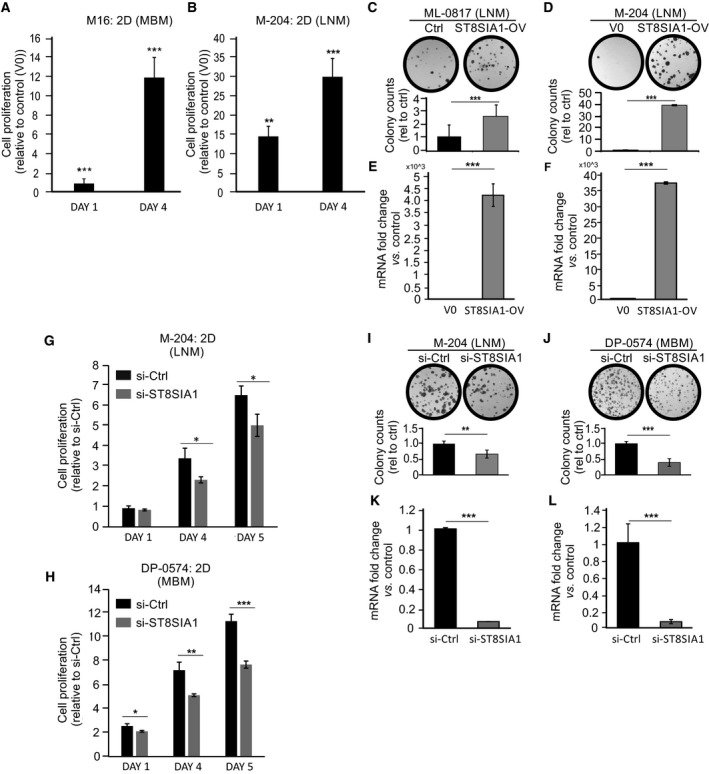
Overexpression and knockdown of ST8SIA1 in melanoma cells. (A, B) Transient ST8SIA1‐overexpression (ST8SIA1‐OV) in cells M16 (A) and M‐204 (B) by transfection with T7‐tagged ST8SIA1 vector or control empty vector (V0). After transfection, cells were seeded in a 2D culture 96‐well plates and at the specified time point cell proliferation was assessed by CellTiter‐Glo. Fold change of ST8SIA1‐OV cells versus control (V0) is shown. (C, D). Colony formation assay on ST8SIA1‐OV and control vector (V0) in ML‐0817 (C) and M‐204 (D) cells. (E, F). ST8SIA1‐OV in ML‐0817 (E) and M‐204 (F) were assessed by qRT‐PCR. (G, H). M‐204 (G) and DP‐0574 (H) cells were transfected with control siRNA (si‐Ctrl) or siRNA for ST8SIA1 (si‐ST8SIA1) and cell proliferation was assessed at specified time point by CellTiter‐Glo. (I, J). Colony formation ability was evaluated in ST8SIA1 KD (si‐ST8SIA1) and control siRNA (si‐Ctrl) M‐204 (I) and DP‐0574 (J). (K, L). ST8SIA1 expression after KD in M‐204 (K) and DP‐0574 (L) was assessed and confirmed by qRT‐PCR. Error bars represent the means ± SD from replicates (*n* = 3) (*t*‐test, *P < 0.05, **P < 0.01, ***P < 0.001).

To characterize the functional effect of ST8SIA1 expression, we overexpressed ST8SIA1 in M16 (MBM), M‐204 (LNM), and ML‐0817 (LNM) cell lines and assessed the effect on cell proliferation. To assess this, we evaluated cell proliferation and colony formation ability in cells with ST8SIA1 overexpression (ST8SIA1‐OV) compared to cells transfected with the control empty vector. Cells with ST8SIA1‐OV grew more rapidly than control cells in culture (Fig. 3A,B). Similarly, the number of colonies increased in cells with ST8SIA1‐OV compared to control cells (Fig. 3C,D), which corresponded with an increased ST8SIA1 expression as corroborated by qRT‐PCR (Fig. 3E,F). Conversely, we knockdown (KD) ST8SIA1 by transfection with ST8SIA1 siRNA pool using DP‐0574 (MBM) and M‐204 (LNM) cell lines. Downregulation of ST8SIA1 inhibited melanoma cell proliferation in cultures (Fig. 3G,H). Similarly, the number of colonies was reduced in ST8SIA1 KD cells compared to control cells (Fig. 3I,J), which corresponded with a decreased ST8SIA1 expression as corroborated by qRT‐PCR (Fig. 3K,L). In summary, high ST8SIA1 expression is associated with increased proliferation and enhanced colony formation ability, whereby downregulation of ST8SIA1 had the reverse effect.

### 3.6 GD3 expression is enhanced in 3D Vitrogel cell cultures mimicking *in vivo* environment

**Fig. 4:**
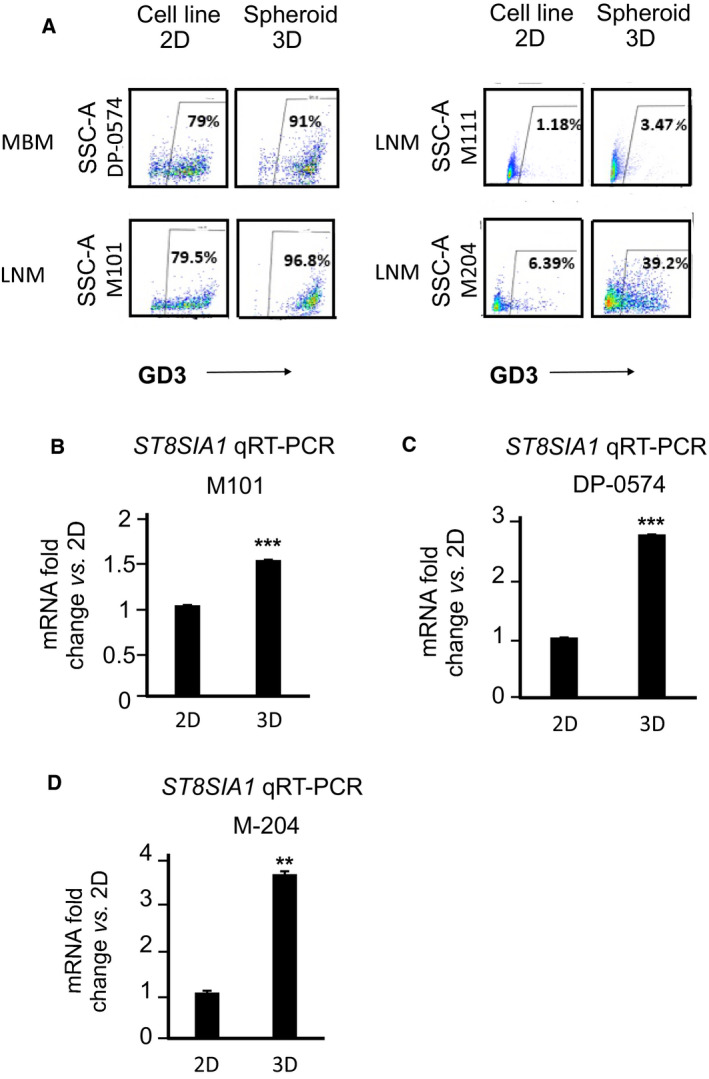
MBM in 3D culture express higher levels of ST8SIA1 and GD3 than those in 2D culture. (A). The percentages of live cells (DP‐0574, M101, M111, M‐204) expressing GD3. Cells were grown in 2D and 3D hydrogel culture, harvested, and phenotypic GD3 expression was assessed by FACS. Cell lines were gated according to live population of cells using 7‐AAD. Within live cell population, GD3 was gated. (B–D). ST8SIA1 mRNA expression in 3D and 2D cultures, relative to the mean expression in 2D culture. Cells (M101, DP‐0574, and M‐204) were grown in 2D culture plates and 3D hydrogel culture, harvested, RNA was isolated, and ST8SIA1 gene expression was assessed by qRT‐PCR. Error bars represent the means ± SD from replicates (*n* = 3) (*t*‐test, NS: not significant, **P < 0.01, ***P < 0.001).

To determine whether GD3 expression is altered when put in the 3D culture hydrogel matrix system for 3 days, which mimics the *in vivo* extracellular matrix microenvironment, we assessed two low GD3 (< 50%): M111 (LNM), and M‐204 (LNM) and also, two high to moderate GD3 (50–90%): DP‐0574 (MBM) and M101 (LNM). Melanoma cell lines growing in 2D cell culture (culture plates) were compared to the respective paired cell lines grown in 3D culture. The GD3 expression increased from 2D to 3D cultures in all four lines assessed (Fig. 4A). Overall, MBM cells consistently grew more successfully compared to LNM in 3D culture. (Fig. S3A). Consistently, we also observed increased ST8SIA1 mRNA expression in those cells with higher GD3 levels, when respective cell lines were cultured in 3D compared to 2D culture conditions (Fig. 4B–D). Then, we determined whether ST8SIA1‐OV increased cell proliferation in 3D conditions. M‐16 (MBM) and M‐204 (LNM) ST8SIA1‐OV cells had enhanced cell proliferation in 3D culture compared to cells transfected with the control empty vector (V0) (Fig. S3B,C). Of note, stable clones overexpressing ST8SIA1 showed an increase in cell surface GD3 expression compared to control (V0) as demonstrated by cell surface immunofluorescence staining (Fig. S4A,B). The increase of GD3 expression from control (V0) to overexpressing ST8SIA1 cells (ST8SIA1‐OV) was more prominent in M‐204 cell line (LNM) (Fig. S4B) than DP‐0574 cell line (MBM) (Fig. S4A), in which the baseline GD3 expression was already high. The enhancement of GD3 was prominent in the cells with strong clusters of GD3 on the cell surface as clearly observed in the overexpressing ST8SIA1 cells (ST8SIA1‐OV). The ST8SIA1 overexpression was confirmed by western blot (Fig. S4C,D). This further confirmed the positive correlation of ST8SIA1 to GD3 expression and that GD3 expression is higher on MBM than on LNM cells.

### 3.7 Icaritin regulates ST8SIA1 expression by targeting p50 in MBM cells

**Fig. 5:**
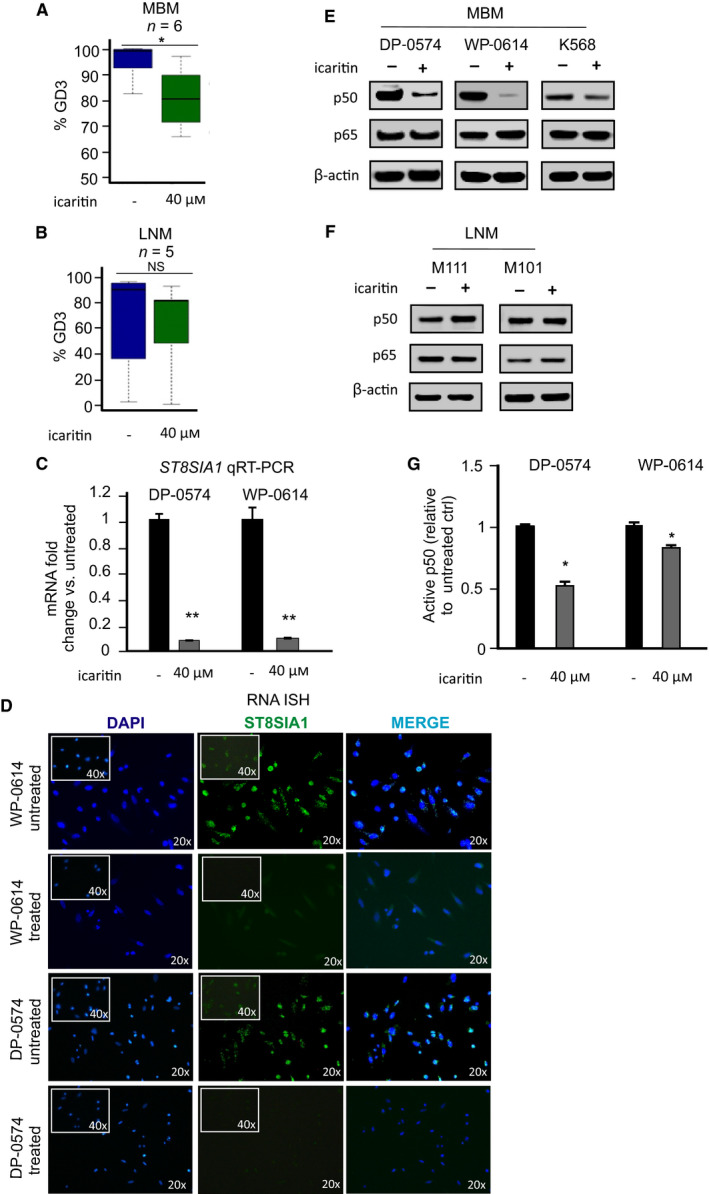
Assessment of GD3 and ST8SIA1 expression after icaritin treatment of MBM cells. (A, B). MBM (*n* = 6) and LNM (*n* = 5) cells were treated 72 h with or without icaritin (40 µm). After 72‐h treatment incubation, cells were harvested and GD3 expression was assessed in MBM (A) and LNM (B) cells by FACS. (C). ST8SIA1 expression was determined by qRT‐PCR in untreated and icaritin‐treated cells. (D). Cells were seeded into and grown in 8‐well chambered slides overnight. Cells were fixed and stained for ST8SIA1 using immunofluorescent probes by RNA‐ISH. ST8SIA1 expression in untreated versus icaritin‐treated cell lines is shown. (E, F). Cells were treated with icaritin (40 µm) or left untreated for 72 h. Cells were harvested, and total protein was extracted. The p50 and p65 levels in MBM (E) and LNM (F) cells were assessed by western blot. (G). Cells were treated with or without icaritin (40 µm) for 72 h, and nuclear extracts were isolated. Transactivation assay was performed to detect p50 active form. Error bars represent means ± SD from replicates (*n* = 3) (*t*‐test, NS: not significant, *P < 0.05, **P < 0.01, ***P < 0.001).

Icaritin, a phytoestrogen and a natural compound derived from *Herba epimedeii*, is previously known to possess potential anticancer activities (Wu *et al*., 2016). Previously, our group showed that icaritin suppresses melanoma growth and modifies fatty acids (Wu *et al*., 2016). Icaritin is currently in clinical trials for HCC (see clinicaltrials.gov). Therefore, we assessed for a potential role of icaritin in controlling ST8SIA1 expression in melanoma cells. We established an optimal dose for icaritin treatment that is not toxic to cells. We found that icaritin reduced cell proliferation in a dose‐dependent manner in MBM and LNM cells using 2D culture (Fig. S5A,B). The effect of icaritin treatment on cell proliferation was then assessed on cell lines grown in 3D cultures. In general, icaritin was effective in suppressing cell proliferation in both MBM and LNM cells (Fig. S5C–F). Moreover, we then assessed the effects of icaritin on the expression of the gangliosides on MBM (*n* = 6) and LNM (*n* = 5) cell lines. Consistently, icaritin treatment of MBM (high GD3) lines significantly decreased GD3 compared to untreated cells (Fig. 5A, Fig. S6A), but did not significantly change the LNM (variable low GD3) lines (Fig. 5B). Moreover, a significant decrease in ST8SIA1 expression was observed in icaritin‐treated versus untreated cells by qRT‐PCR and RNA‐ISH (Fig. 5C,D, respectively). Overall, icaritin downregulated ST8SIA1 and GD3 expression specifically in MBM with a high GD3 expression indicating a phenotypic indicator of icaritin responsiveness.

Recently, we have shown the significant role of RNF123 in NF‐κB regulation in melanoma distant metastasis as related to p50 production (Iida *et al*., 2017; Kravtsova‐Ivantsiv *et al*., 2015). P50 dimers and p50 and p65 heterodimers play a significant role in NF‐κB activity of targeted genes promoter region (Iida *et al*., 2017). As rapid activation of the NF‐κB pathway has been suggested to enhance ST8SIA1 and GD3 expression in Fas‐induced Jurkat T cells (Kang *et al*., 2006), we examined the levels of p50 and p65 in icaritin‐treated melanoma LNM and MBM cell lines compared to untreated cells. Our results revealed p65 levels were not altered after icaritin treatment; however, p50 was significantly reduced in MBM cells (Fig. 5E), but not in LNM cells (Fig. 5F) treated with icaritin. The decrease of p50 in MBM cells after icaritin treatment was further corroborated by the decrease of ST8SIA1 mRNA expression in MBM cells (Fig. 5C). In transactivation assays, we monitored the nuclear translocation of p50 as indicative of active p50. Concordantly, icaritin treatment diminished the amount of p50 translocated into the nucleus in MBM cells (Fig. 5G). Our results suggested that icaritin inhibited NF‐κB‐mediated activation of ST8SIA1 by downregulating p50, thus reducing p50 active form in MBM cells, thereby reducing ST8SIA1 expression and subsequently cell surface GD3 (Fig. S7A,B).

## 4 Discussion

The major findings of the study are that ST8SIA1 upregulation is a key metabolic enzyme, which promotes GD3 expression in MBMs, and may play a role in MBM development and progression. ST8SIA1 expression is variable in LNM, whereby its enhanced expression in LNM is a significant prognostic factor of poor disease outcome. GD3 cell surface expression is a phenotype that favors successful MBM growth. Icaritin treatment decreases p50 expression and downregulates ST8SIA1 and GD3 expression by decreasing p50 expression, a major activator of ST8SIA1. GD3 serves as a phenotypic indicator of icaritin responsiveness.

We demonstrated, for the first time, a significantly higher expression of ST8SIA1 in MBM compared to LNM and PRM. Moreover, as the ganglioside pathway preferentially leads to GD3 expression through increased ST8SIA1 activity in highly advanced metastatic melanomas, such as MBM, the expression levels of GM3 and GM2 were decreased compared to LNM. Although we did not see significant difference in the other major disialic GD2, its expression is low in metastatic tumors likely due to the decrease of the synthase β4GALNT1 which converts GD3 to GD2 (Tsuchida *et al*., 1987b). This could also explain the elevation of GD3. Moreover, GD2 is antigenic, which is known to induce anti‐GD2 IgM elevation in melanoma patients (Tai *et al*., 1985; Takahashi *et al*., 1999); therefore, GD2+ melanoma cells may be eliminated in patients.

O‐acetylated GD3 is found on some melanoma cells, whereby it is derived from GD3 as an alternative downstream pathway instead of GD2 synthesis (Ravindranath *et al*., 1991). Its synthesis is from precursor GD3 through the enzyme, sialate *O*‐acetyltransferase (CasD1), which adds acetyl groups to sialic acid (Baumann *et al*., 2015). Previous studies by Ravindranath *et al*., Cheresh *et al*, Thurin J *et al*, have shown that O‐acetylated GD3 is present in melanoma cells (Cheresh *et al*., 1984; Ravindranath *et al*., 1991; Thurin *et al*., 1986); however, the level is very low overall, which represents a low percentage of the total lipid‐bound sialic acid, and not significant as compared to GD3 (Ravindranath *et al*., 1991). O‐acetylated GD3 function and role of cell surface expression still not well understood.

The upregulation of the enzyme ST8SIA1 in a specific subset of LNM and MBM indicates its potential role in aggressive growth of regional metastasis and supports MBM development, leading eventually to poor outcomes. This observation is consistent with our previous findings that PRM and LNM enriched in GM3 grow slower and show more favorable prognosis (Tsuchida *et al*., 1989). Moreover, decreasing ST8SIA1 expression by siRNA‐mediated KD suppressed melanoma cell proliferation. Hence, the GM3 active metabolism pathway to GD3 through ST8SIA1 may represent an environment phenotype promoting brain metastasis development and LNM aggressiveness to metastasize. Important differences in ganglioside phenotype of melanoma were observed in 2D and 3D cultures, whereby 3D culture significantly had developed enhanced GD3 levels for GD3 low‐expressing metastatic cells in respective 2D cultures. Overall, GD3 expression changes in *in vitro* 2D cultures versus *in vivo* tumors reiterates the caution needed in characterization of melanoma cell phenotype changes in 2D cell culture which have a plastic attachment environment influence versus clinical tumor environment.

GD3 has been shown to play a role in adhesion, signal transduction, cell‐to‐cell communication, neural development, and pathogenesis in brain‐related diseases such as Huntington's disease, in which elevated GD3 levels have been reported (Yu *et al*., 2012). GD3 functional roles in cutaneous melanomas are not well understood. Previous studies have shown that the presence of GD3 is correlated with stronger extracellular matrix (ECM) adhesion and focal adhesion kinase tyrosine phosphorylation (Ohkawa *et al*., 2010). This may explain the activation of ST8SIA1 in ECM hydrogel 3D culture. A previous study demonstrated that GD1α ganglioside was involved in breast cancer brain metastasis supporting the migration of breast cancer cells through the blood–brain barrier (Bos *et al*., 2009); however, in metastatic cutaneous melanoma, GD1α is not significantly expressed. Melanoma gangliosides like GD3, the predominant ganglioside expressed in metastatic melanoma, are known to promote tumor migration (Merzak *et al*., 1995) and angiogenesis (Ziche *et al*., 1992). Moreover, GD3‐positive cells activate paxillin, p130Cas, and focal adhesion kinase, promoting tumor cell growth, and invasion (Hamamura *et al*., 2008). GD3 has been shown to have a significant role in metastasis (Liu *et al*., 2018). Further studies will help elucidate its role. In addition, our mouse MBM model presented in this study showed that the melanoma brain‐metastasizing variant has higher ST8SIA1 level compared to the respective cutaneous variant, supporting the GD3 role in melanoma cell migration, metastasis to the brain, and penetration through the blood–brain barrier.

GD3‐rich lipid rafts can also modulate the MAPK signaling pathway, inducing cell proliferation and self‐renewal of neural stem cells (Yu *et al*., 2012). The MAPK signaling pathway is highly active in advanced metastatic melanomas, particularly through the BRAF pathway (Amaral *et al*., 2017; Davies *et al*., 2002). Enhanced GD3 expression on melanoma cells may be an advantage in the brain microenvironment niche. GD3 plays a role in self‐renewal (Yu *et al*., 2012) and may be neuroprotective via antiapoptotic effects, or inhibition of immune infiltration, and/or promoting survival and metastasis. In MBM tumors, the expression of ST8SIA1 appears in patches in some specimens, as shown by RNA‐ISH, suggesting that ST8SIA1 expression could be enhanced in the regenerative niche and explaining tumor heterogeneity in MBM. Also, our studies demonstrate the significance of GD3 expression in LNM that are aggressive and in developing distant melanoma metastasis. LNM and visceral metastasis GD3 were not consistently high levels as in MBM.

Icaritin was found to downregulate ST8SIA1 and concurrently cell surface GD3 expression. It is currently in clinical trials for treatment of hepatocellular carcinoma (HCC) (Phase II), metastatic breast cancer (Phase I), and other solid tumors (clinicaltrials.gov). Icaritin has been demonstrated to be a safe agent in patients and efficient therapeutic as previously shown in a Phase II clinical trial for HCC patients (Sun *et al*., 2018). Recently, icaritin has been shown to be effective in combination with pembrolizumab in HCC patients as well (Fan *et al*., 2019). Icaritin has potential to be used in other cancers such as in metastatic melanoma. Our group assessed another known natural compound affecting ganglioside synthesis, triptolide (a diterpenoid epoxide), which can inhibit GD3 synthase and melanoma cell proliferation (Kwon *et al*., 2010), as a control to icaritin effect on ganglioside synthesis. However, triptolide‐treated melanoma cell lines did not show a significant changes in colony formation compared to icaritin‐treated cells, whereby reduction of GD3 expression was only found in one cell line (MBM) out of 6 melanoma cell lines (4 MBM and 2 LNM) assessed. These results suggested that triptolide may exhibit different metabolic and growth pathway regulation compared to icaritin and acts on select melanoma cell lines, and/or may also depend on individual cell drug uptake.

## 5 Conclusion

ST8SIA1 as a theranostic target for MBM has potential clinical importance as there are limited effective treatments for MBM. Icaritin‐mediated downregulation of p50 offers new strategies for targeting ST8SIA1 to reduce melanoma aggressiveness. In conclusion, our results suggest that ST8SIA1 expression leads to elevated cell surface GD3 expression, which is a phenotype that associates with MBM development and progression. Furthermore, increased ST8SIA1 levels are associated with poor prognosis in LNM and can potentially be targeted by icaritin.

## Conflict of interest

The authors declare no conflicts of interest.

## Author contributions

RIR, MAB, JW, and DSBH conceived and designed the study. RIR and MAB involved in development of methodology. RIR, MAB, SI, OS‐A, IPW, SLS, RFI, and DFK acquired the data (provided animals, acquired and managed patients, provided facilities, abs, clinical brain specimens, etc.). RIR, MAB, SCC, SLS, GBM, MAD, and DSBH analyzed and involved in interpretation of data (e.g., statistical analysis, biostatistics, computational analysis). RIR, MAB, JW, PJ, EK, XZ, SCC, SLS, SI, IPW, RFI, and DSBH wrote, reviewed, and/or revised the manuscript. RIR, MAB, GBM, MAD, KT, XZ, EK, PJ, and DSBH made administrative, technical, or material support (i.e., reporting or organizing data, constructing databases). DBSH supervised and funded the study.

## Supporting information


**Fig. S1.** ST8SIA1 expression correlates with GD3 expression in LNM and MBM. (A). Cells were seeded, fixed and hybridized with ST8SIA1 probe. Immunofluorescence staining of ST8SIA1 (green), DAPI (blue) and merged for LNM and MBM cell lines using RNA‐ISH are shown. (B). Correlation analysis between ST8SIA1 expression (assessed by qRT‐PCR) and the cell surface gangliosides GM3 and GD3 (assessed by FACS). ST8SIA1 is positively correlated with GD3 (r=0.90) and negatively correlated with GM3 (r=‐0.16). (C). ST8SIA1 mRNA expression was assessed by qRT‐PCR on MBM frozen tissues (n=10) and compared to benign tissues: meningiomas (n=5) and benign pituitary disease (n=5) frozen tissues. Meningioma and benign pituitary frozen tissues (n=10) were used as a control for MBM frozen tissue analysis of ST8SIA1. Bars show the mean fold change of MBM frozen tissues versus meningioma. Error bars represent SEM (t‐test; *p<0.05).

**Fig. S2.** Relationship of ST8SIA1 expression and clinical variables using TCGA dataset. (A). ST8SIA1 expression in PRM, LNM, and distant organ metastasis (DOM). (B). ST8SIA1 expression in Stage I‐II and III‐IV PRM tumors. (C). ST8SIA1 expression in PRM with Ulceration (YES) or no Ulceration (NO), (D). Breslow depth of PRM. (E). Association between ST8SIA1 expression and age at diagnosis. (F). ST8SIA1 expression in melanoma patients with different mutations: BRAF mutated; NF1 mutated; NRAS mutated; or triple WT (no mutation in BRAF, NF1 or NRAS).

**Fig. S3.** Analysis of cell proliferation and colony formation of melanoma lines with ST8SIA1‐enhanced expression. (A). Cell proliferation analysis in LNM and MBM cell lines grown in 3D culture conditions. (B‐C). ST8SIA1‐overexpressing (ST8SIA1‐OV) cells M16 (B) and M‐204 (C) were established by transfection with T7‐tagged ST8SIA1 vector or empty control vector (V0) as a control. After transfection, cells were seeded in spheroid cultures. Melanoma cell proliferation was assessed by luminescent cell viability assay for days 1, 5, and 10 for M16 and days 1, 4, and 5 for M‐204. Representative photos of spheroid empty vector (V0) and ST8SIA1‐overexpressing (ST8SIA1‐OV) cells are shown.

**Fig. S4.** GD3 expression in ST8SIA1 overexpressing cell lines. (A‐D). Stable clones overexpressing ST8SIA1 (ST8SIA1‐OV) or the empty vector (V0) were established. Cells were seeded and stained for GD3. Images of immunofluorescence staining patterns of GD3 (red), DAPI (blue) and merge for DP‐0574 (MBM) (A) and M‐204 (LNM) (B) cell lines are shown (Scale bars: 25 µm). Overexpression of ST8SIA1 was confirmed by western blot for DP‐0574 (C) and M‐204 (D).

**Fig. S5.** Treatment with icaritin reduces cell viability and colony formation of melanoma cells in 2D and 3D cultures. (A‐B). Cell lines MBM (A) and LNM (B) were grown in a 2D culture in a 96‐well plate and either treated with icaritin (40 µM or 80 µM) or left untreated. Cell viability was assessed after 3 days of culture by CellTiter‐Glo. (C‐F). Cell lines treated with icaritin (40 µM) or left untreated were grown in a 3D culture in spheroid 96‐well plate for 3 days. Photos of the spheroid formation by MBM (C) and LNM (E) untreated and icaritin‐treated cells taken at days 1 and 3 are shown (Scale bars = 100 µm). Cell viability of MBM (D) and LNM (F) cultures were assessed after 4 days of culture by CellTiter‐Glo. Error bars represent means ± SD from replicates (n=3) (t‐test; NS=not significant, **p<0.01, ***p<0.001).

**Fig. S6.** Ganglioside profile on melanoma cell lines after icaritin. (A). MBM cells (K568 and WP‐0614) were not treated (untreated) or treated with icaritin (40 µM) for 72h and then assessed by FACS. Cell lines were gated according to live population of cells using 7‐AAD. Within live population of cells, GD3‐ positive cells were gated.

**Fig. S7.** Schematic representation of ST8SIA1 and cell surface GD3 regulation through NF‐κB pathway. (A). In MBM cells, p50/p50 homodimers (transcription repressor) have reduced effect whereby p50/p65 heterodimers (tumor promoter) are enhanced and drive activation of NF‐κB targeted genes such as ST8SIA1. Active p50/p65 heterodimers translocate into the nucleus promoting ST8SIA1 expression, and consequently exacerbating cell surface GD3 expression and enhancing cell proliferation. (B). Icaritin treatment of MBM cells significantly reduces p50 and its downstream interactions such as p50/p50 homodimer and p50/p65 heterodimer; reducing ST8SIA1 and cell surface GD3 expression and suppressing cell proliferation.
