# Supplementary figures and images for: Upregulation of cell surface GD3 ganglioside phenotype is associated with human melanoma brain metastasis
Source: Mol Oncol. 2020 Jun 5;14(8):1760–78. doi: 10.1002/1878-0261.12702 (PMC7400791; doi:10.1002/1878-0261.12702)

**Supplementary Figure S1**

**A. RNA ISH**

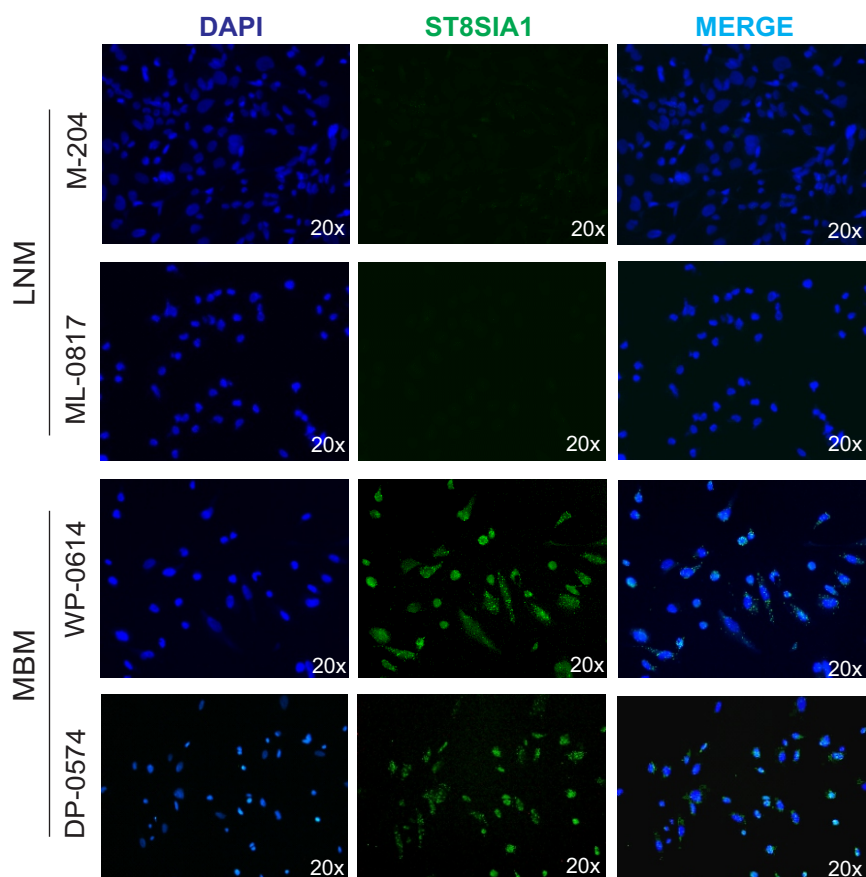

**B.**

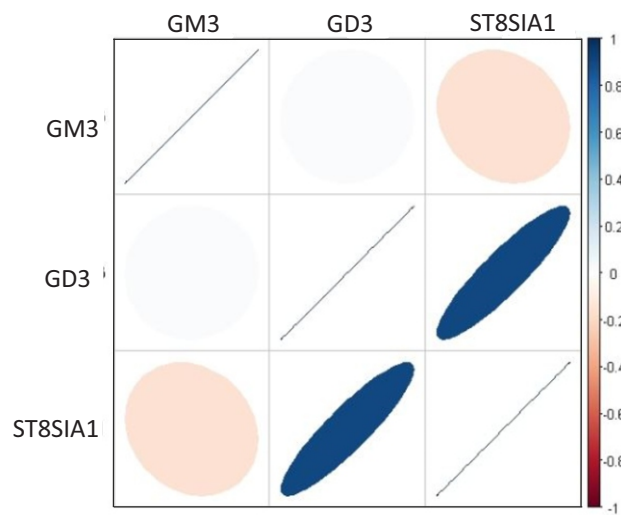

**C.**

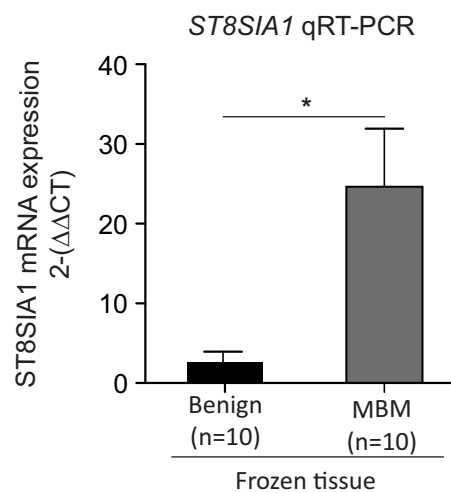

Supplement: Supplementary file 1 — Fig. S1. ST8SIA1 expression correlates with GD3 expression in LNM and MBM. (A). Cells were seeded, fixed and hybridized with ST8SIA1 probe. Immunofluorescence staining of ST8SIA1 (green), DAPI (blue) and merged for LNM and MBM cell lines using RNA‐ISH are shown. (B). Correlation analysis between ST8SIA1 expression (assessed by qRT‐PCR) and the cell surface gangliosides GM3 and GD3 (assessed by FACS). ST8SIA1 is positively correlated with GD3 (r=0.90) and negatively correlated with GM3 (r=‐0.16). (C). ST8SIA1 mRNA expression was assessed by qRT‐PCR on MBM frozen tissues (n=10) and compared to benign tissues: meningiomas (n=5) and benign pituitary disease (n=5) frozen tissues. Meningioma and benign pituitary frozen tissues (n=10) were used as a control for MBM frozen tissue analysis of ST8SIA1. Bars show the mean fold change of MBM frozen tissues versus meningioma. Error bars represent SEM (t‐test; *p<0.05). [file MOL2-14-1760-s001.pdf]

Supplementary Figure S2

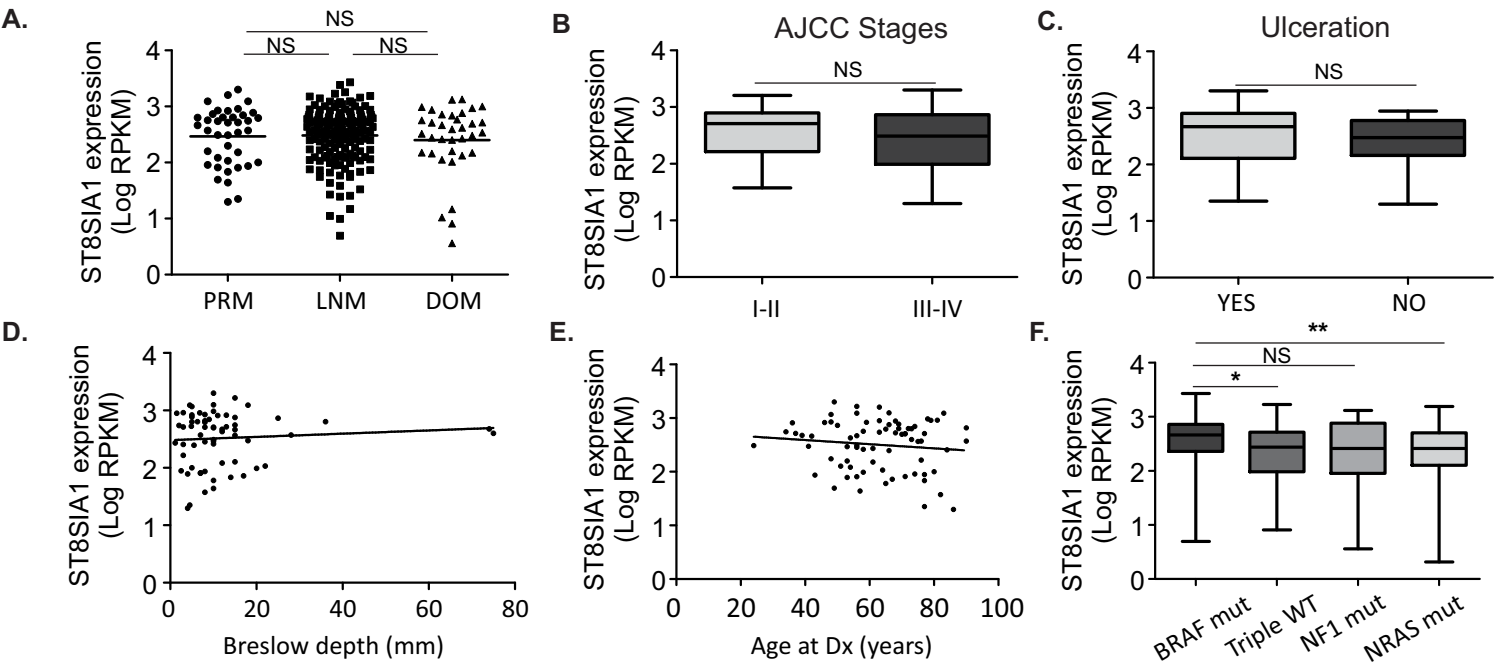

Supplement: Supplementary file 2 — Fig. S2. Relationship of ST8SIA1 expression and clinical variables using TCGA dataset. (A). ST8SIA1 expression in PRM, LNM, and distant organ metastasis (DOM). (B). ST8SIA1 expression in Stage I‐II and III‐IV PRM tumors. (C). ST8SIA1 expression in PRM with Ulceration (YES) or no Ulceration (NO), (D). Breslow depth of PRM. (E). Association between ST8SIA1 expression and age at diagnosis. (F). ST8SIA1 expression in melanoma patients with different mutations: BRAF mutated; NF1 mutated; NRAS mutated; or triple WT (no mutation in BRAF, NF1 or NRAS). [file MOL2-14-1760-s002.pdf]

Supplementary Figure S3

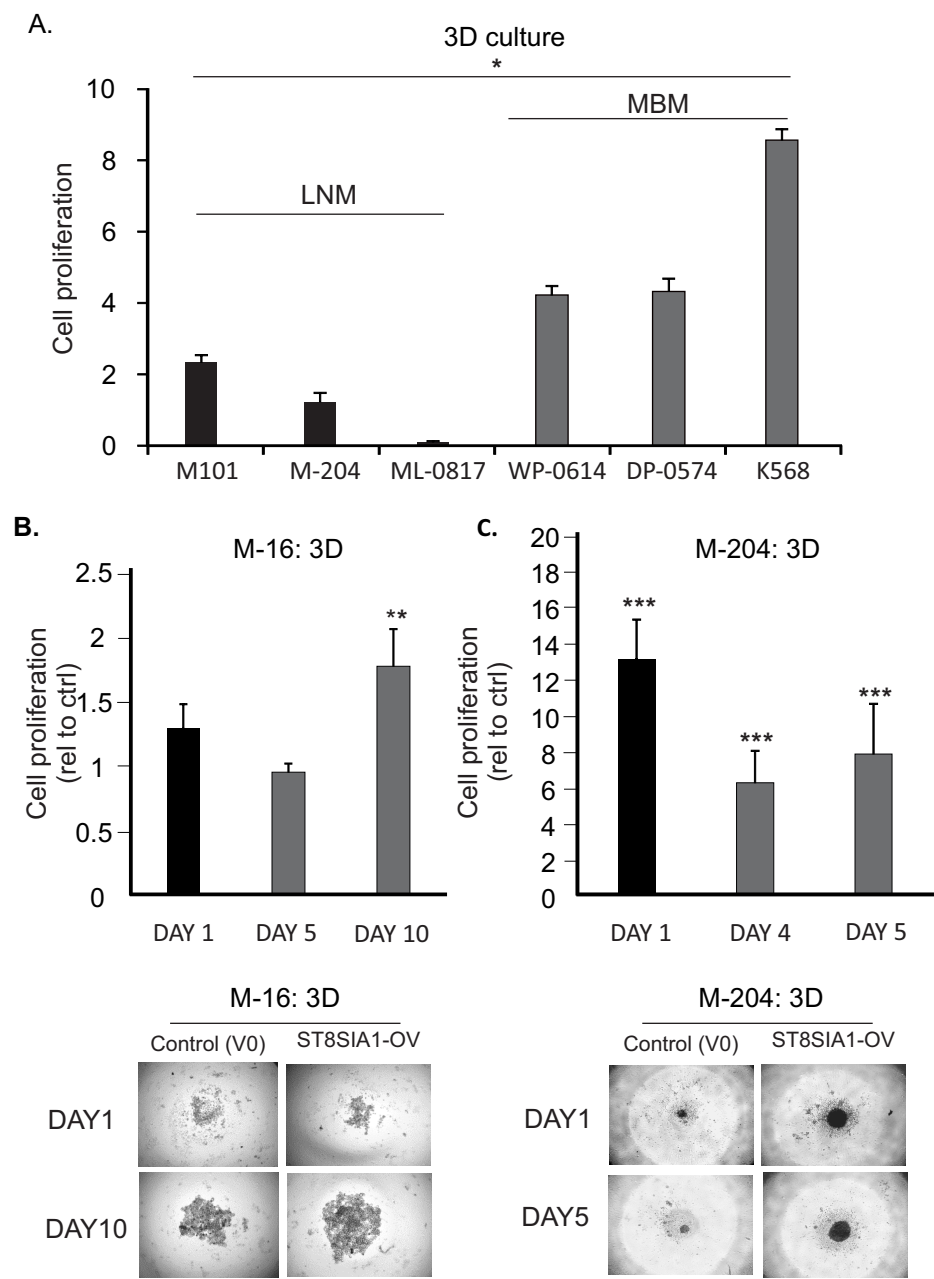

Supplement: Supplementary file 3 — Fig. S3. Analysis of cell proliferation and colony formation of melanoma lines with ST8SIA1‐enhanced expression. (A). Cell proliferation analysis in LNM and MBM cell lines grown in 3D culture conditions. (B‐C). ST8SIA1‐overexpressing (ST8SIA1‐OV) cells M16 (B) and M‐204 (C) were established by transfection with T7‐tagged ST8SIA1 vector or empty control vector (V0) as a control. After transfection, cells were seeded in spheroid cultures. Melanoma cell proliferation was assessed by luminescent cell viability assay for days 1, 5, and 10 for M16 and days 1, 4, and 5 for M‐204. Representative photos of spheroid empty vector (V0) and ST8SIA1‐overexpressing (ST8SIA1‐OV) cells are shown. [file MOL2-14-1760-s003.pdf]

Supplementary Figure S4

A. Immunofluorescence

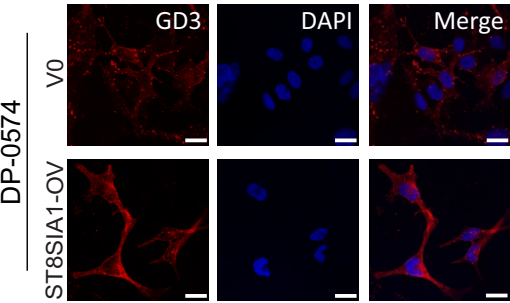

B.

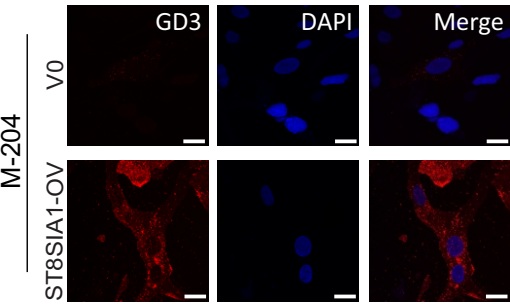

C.

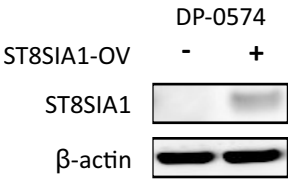

D.

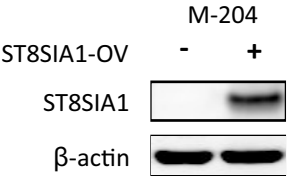

Supplement: Supplementary file 4 — Fig. S4. GD3 expression in ST8SIA1 overexpressing cell lines. (A‐D). Stable clones overexpressing ST8SIA1 (ST8SIA1‐OV) or the empty vector (V0) were established. Cells were seeded and stained for GD3. Images of immunofluorescence staining patterns of GD3 (red), DAPI (blue) and merge for DP‐0574 (MBM) (A) and M‐204 (LNM) (B) cell lines are shown (Scale bars: 25 µm). Overexpression of ST8SIA1 was confirmed by western blot for DP‐0574 (C) and M‐204 (D). [file MOL2-14-1760-s004.pdf]

Supplementary Figure S5

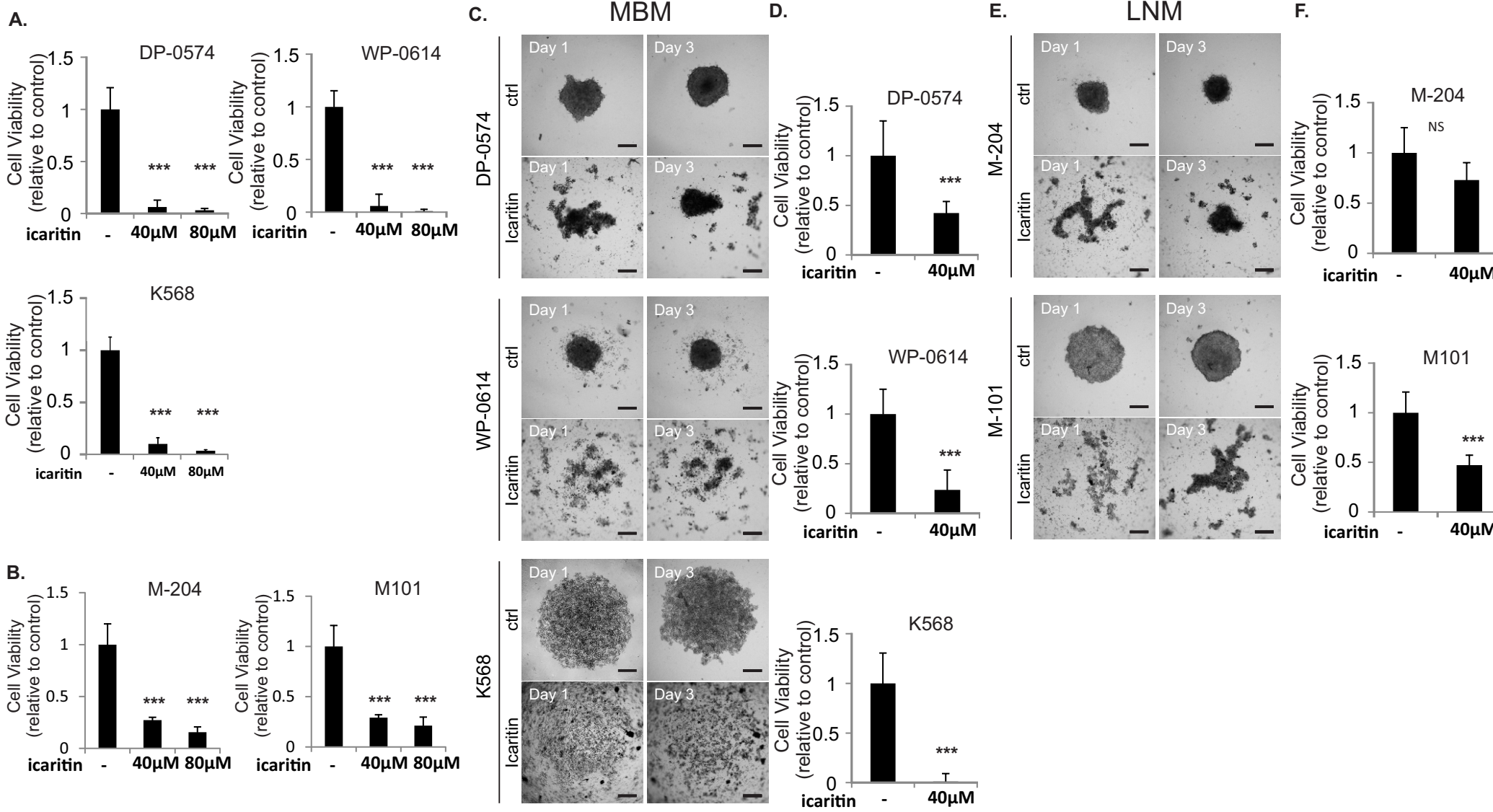

Supplement: Supplementary file 5 — Fig. S5. Treatment with icaritin reduces cell viability and colony formation of melanoma cells in 2D and 3D cultures. (A‐B). Cell lines MBM (A) and LNM (B) were grown in a 2D culture in a 96‐well plate and either treated with icaritin (40 µM or 80 µM) or left untreated. Cell viability was assessed after 3 days of culture by CellTiter‐Glo. (C‐F). Cell lines treated with icaritin (40 µM) or left untreated were grown in a 3D culture in spheroid 96‐well plate for 3 days. Photos of the spheroid formation by MBM (C) and LNM (E) untreated and icaritin‐treated cells taken at days 1 and 3 are shown (Scale bars = 100 µm). Cell viability of MBM (D) and LNM (F) cultures were assessed after 4 days of culture by CellTiter‐Glo. Error bars represent means ± SD from replicates (n=3) (t‐test; NS=not significant, **p<0.01, ***p<0.001). [file MOL2-14-1760-s005.pdf]

Supplementary Figure S6

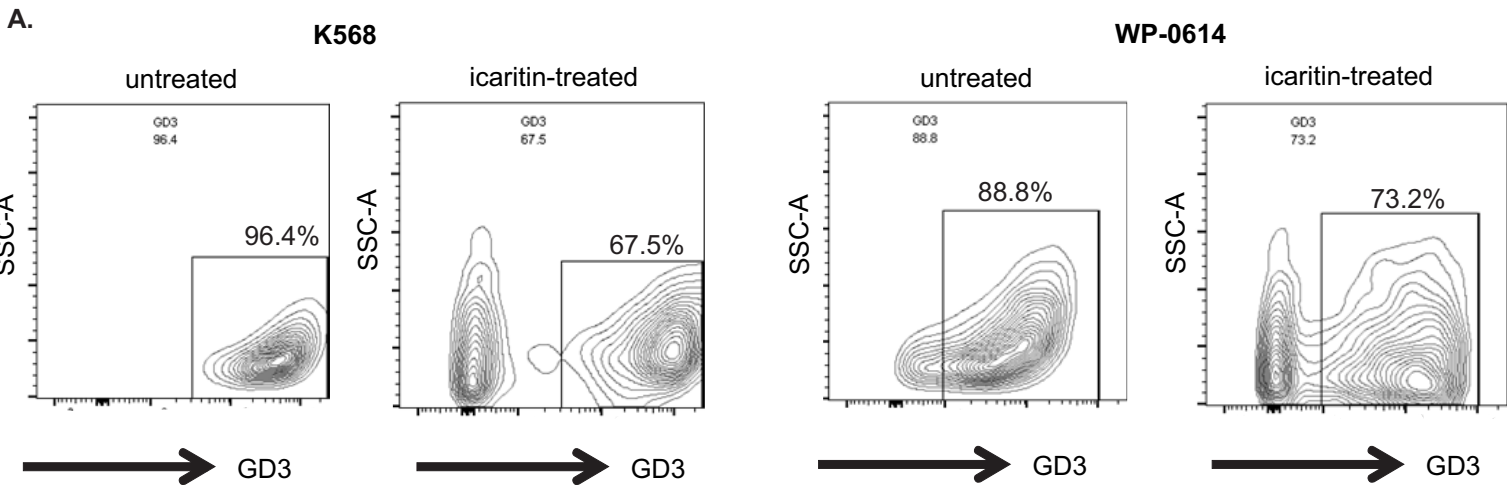

Supplement: Supplementary file 6 — Fig. S6. Ganglioside profile on melanoma cell lines after icaritin. (A). MBM cells (K568 and WP‐0614) were not treated (untreated) or treated with icaritin (40 µM) for 72h and then assessed by FACS. Cell lines were gated according to live population of cells using 7‐AAD. Within live population of cells, GD3‐ positive cells were gated. [file MOL2-14-1760-s006.pdf]

Supplementary Figure S7

A. MBM (High ST8SIA1)

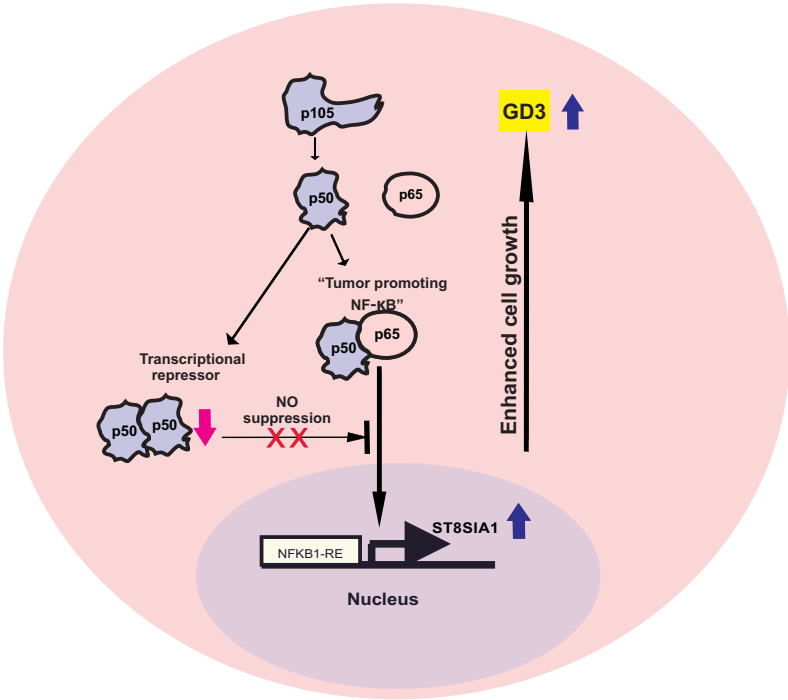

B. MBM + icaritin

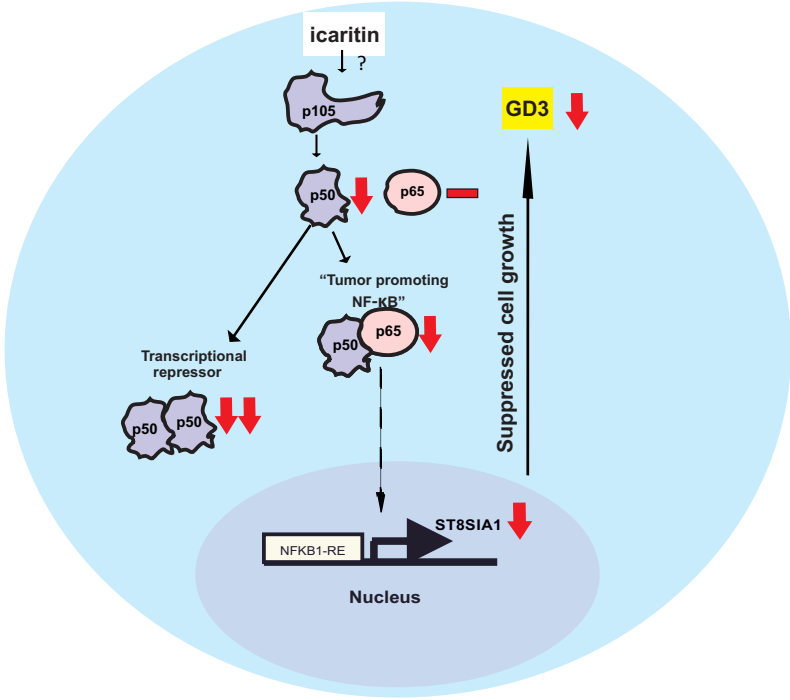

Supplement: Supplementary file 7 — Fig. S7. Schematic representation of ST8SIA1 and cell surface GD3 regulation through NF‐κB pathway. (A). In MBM cells, p50/p50 homodimers (transcription repressor) have reduced effect whereby p50/p65 heterodimers (tumor promoter) are enhanced and drive activation of NF‐κB targeted genes such as ST8SIA1. Active p50/p65 heterodimers translocate into the nucleus promoting ST8SIA1 expression, and consequently exacerbating cell surface GD3 expression and enhancing cell proliferation. (B). Icaritin treatment of MBM cells significantly reduces p50 and its downstream interactions such as p50/p50 homodimer and p50/p65 heterodimer; reducing ST8SIA1 and cell surface GD3 expression and suppressing cell proliferation. [file MOL2-14-1760-s007.pdf]
